# Risk factors for non-vancomycin-resistant enterococcus faecium infection

**DOI:** 10.1186/2197-425X-3-S1-A124

**Published:** 2015-10-01

**Authors:** R Jiménez, A Ortín, S Rebollo, L Herrera, A Fernández, M Galindo, S Moreno, A Ojados, L Tárraga, Y Bonilla, S Sánchez-Argente, MM Ortíz, MJ Del Amor, JM Allegue

**Affiliations:** Servicio de Medicina Intensiva, Hospital General Universitario Santa Lucía, Cartagena, Spain; Servicio de Microbiología, Hospital General Universitario Santa Lucía, Cartagena, Spain

## Introduction

Enterococci spp. cause serious infections in ICU environment. Among them, Enterococcus faecium may constitute a problem when choosing empirical treatment. In addition, vancomycin-resistant Enterococci is growing as an epidemiological problem.

## Objectives

To identify risk factors for acquisition of an E. Faecium infection in patients admitted to Intensive Care Unit (ICU).

## Methods

We analyzed retrospective data from patients admitted to a polivalent ICU during a 4-year period. We specifically analyzed those patients with an infection during their ICU stay and search for factors related to the isolation of E. faecium from clinical samples.

## Results

We studied 841 patients, 21 of them (2.5 %) with an E. Faecium infection. No E. faecium vancomycin resistance was found.

Patients with E. Faecium isolation were older (69.5 vs 64), infections had been acquired in the hospital, were surgical patients in higher proportion and had longer ICU stay (81 vs 44.3 % p 0.001 and 57.1 vs 27.5 % p 0.003 and 6 (3-11) vs 13 (6-25) p 0.001 respectively), had received parenteral nutrition (43 v 13 % p 0.001) and were malnourished in higher rate (25 v 4.2 % p 0.001) Prior use of piperacilina-tazobactam, carbapenem or linezolid were found more frequently in E. Faecium-infected patients (52.4 vs 24.1 % p 0.003 and 66.7 vs 20.6 % p 0.001) ESBL isolation coexisted more frequently in patients with E. Faecium infection. Mortality rate was higher in patients with E. faecium infection (57 vs 25.6 % p 0.001).

Logistic regression analysis showed nosocomial admission, prior use of piperaciline-tazobactam or carbapenem and ESBL isolation as independent risk factors for E. faecium infection. E. faecium infection was not independently associated with mortality.

## Conclusions

Non-community admissions and prior exposure to piperaciline-tazobactam and carbapenem resulted being associated with isolation of E. faecium in our patients.

ESBL isolation appeared independently associated with E. faecium, probably because both bacteria share ways of selection. Controlling raising incidence of E. faecium may prevent vancomycin-resistant Enterococci spread, not still a problem in our environment.
